# Synchronous Small Bowel Gangrene With Pyelonephritis Secondary to Mucormycosis: A Disastrous Complication of COVID-19 Pandemic

**DOI:** 10.7759/cureus.15911

**Published:** 2021-06-25

**Authors:** Vaibhav K Varshney, Ashish Swami, Balamurugan Thirunavukkarasu, Ashish Agarwal, Gaurav Baid

**Affiliations:** 1 Surgical Gastroenterology, All India Institute of Medical Sciences, Jodhpur, IND; 2 Pathology, All India Institute of Medical Sciences, Jodhpur, IND; 3 Gastromedicine, All India Institute of Medical Sciences, Jodhpur, IND; 4 Urology, All India Institute of Medical Sciences, Jodhpur, IND

**Keywords:** covid-19, vascular necrosis, gastrointestinal mucormycosis, bowel gangrene, angioinvasive

## Abstract

Mucormycosis is a rare infection caused by fungi of the order *Mucorales*. The infection frequently involves the rhino-cerebral or respiratory system and involvement of the gastrointestinal (GI) tract and kidney are rare. It usually infects immunocompromised individuals due to various causes and an upsurge is hypothesized to be linked with irrational use of steroids during coronavirus disease 2019 (COVID-19) pandemic.

We encountered a rare case of systemic mucormycosis that involved both renal as well as mesenteric vessels and led to ischemia of both vital organs. The patient developed massive bowel gangrene involving the duodenum, proximal jejunum, and left kidney due to angioinvasive mucormycosis.

The diagnosis of GI mucormycosis may further increase during the current pandemic. The physicians, as well as surgeons, should be aware of this unwanted complication and keep a high index of suspicion for this rare disease.

## Introduction

Mucormycosis is a rare, potentially fatal fungal infection and generally involves rhino-orbito-cerebral and respiratory tracts. It primarily occurs in immunocompromised hosts with various risk factors [1]. There is an ongoing epidemic of mucormycosis in India which is postulated to be linked with the irrational over-the-counter usage of steroid medications to treat coronavirus infectious disease (coronavirus disease 2019, COVID-19).

Gastrointestinal (GI) and renal mucormycosis are uncommon and simultaneous involvement of both systems are even rare. Diagnosis is difficult and often delayed due to non-specific clinical symptoms or signs and thus bears severe morbidity and mortality [2-3]. We hereby present a case of a young adult male patient of systemic mucormycosis with involvement of both gut and kidney following prolonged corticosteroid intake for COVID-19 infection. This case highlights the need for a high index of suspicion for early detection and adequate management of this catastrophic illness.

## Case presentation

A 32-year-old male patient presented with complaints of high-grade fever, shortness of breath, and pain in the left side of the abdomen for the last one week. He had a history of being infected with COVID-19 around one month back for which he was hospitalized and was managed with oxygen support, injectable steroids, and other supportive management. Subsequently, the patient continued consumption of oral steroids at home without any proper guidance. At admission, the patient was found to have tachycardia and tachypnea with hypotension. He was immediately shifted to ICU and started on IV fluid, broad-spectrum antibiotics, oxygen, and inotropic support.

Ultrasonography of abdomen showed bilateral renal calculi, bulky left kidney with the monophasic flow in the left renal segmental vessels. Further, blood investigations showed raised leucocyte count (32,000/mm3) and deranged renal function (serum creatinine - 1.8 mg/dL). A differential of urinary tract infection with sepsis was kept and a non-contrast computed tomography (NCCT) of the abdomen was performed which showed bulky left kidney with significant perinephric fat stranding suggestive of pyelonephritis. The IV antibiotics were continued and a double ‘J’ stent was placed in the left kidney. Urine cultures, however, did not reveal any bacterial growth.

**Figure 1:**
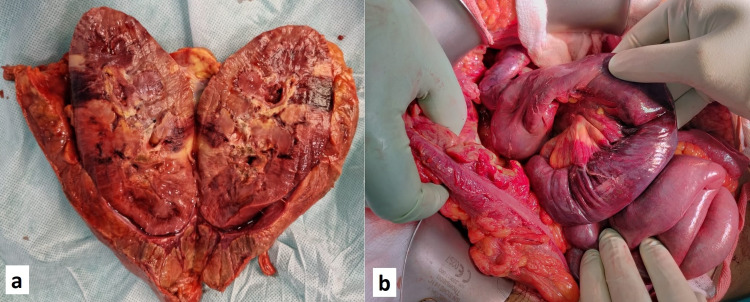
Intra-operative images. a) Resected specimen of bulky left kidney with areas of necrosis; b) Gangrenous bowel along with thickened mesentery

The patient also had an episode of melena after four days of admission and was ascribed to prophylactic low molecular weight heparin, which was subsequently stopped. However, his clinical condition deteriorated rapidly with worsening sepsis, progressive renal dysfunction, and hemodynamic instability. In view of deteriorating clinical condition due to left side pyelonephritis, he underwent left-sided nephrectomy via the retroperitoneal incision. Intra-operatively, post-nephrectomy bowel appeared ischemic and congested. Subsequently, an exploratory laparotomy was performed and bowel necrosis extending from the third part of the duodenum till ~100cm of proximal jejunum was noted. Apart from this, ischemic changes were also confirmed in the transverse and descending colon and mesentery of the affected bowel segment had congestive thickening with large areas of induration and thick whitish discharge (Figure 1A,B). The gangrenous segment of the bowel beginning from the D3 till proximal jejunum was resected and a Stamm’s gastrostomy, feeding jejunostomy from the distal jejunal segment with diverting ileostomy was made. Sub-hepatic and pelvic abdominal drains were placed and the patient was shifted to ICU on inotropic support. However, he developed severe metabolic acidosis, septic shock with anuria and succumbed to his illness after 10 hours.

**Figure 2:**
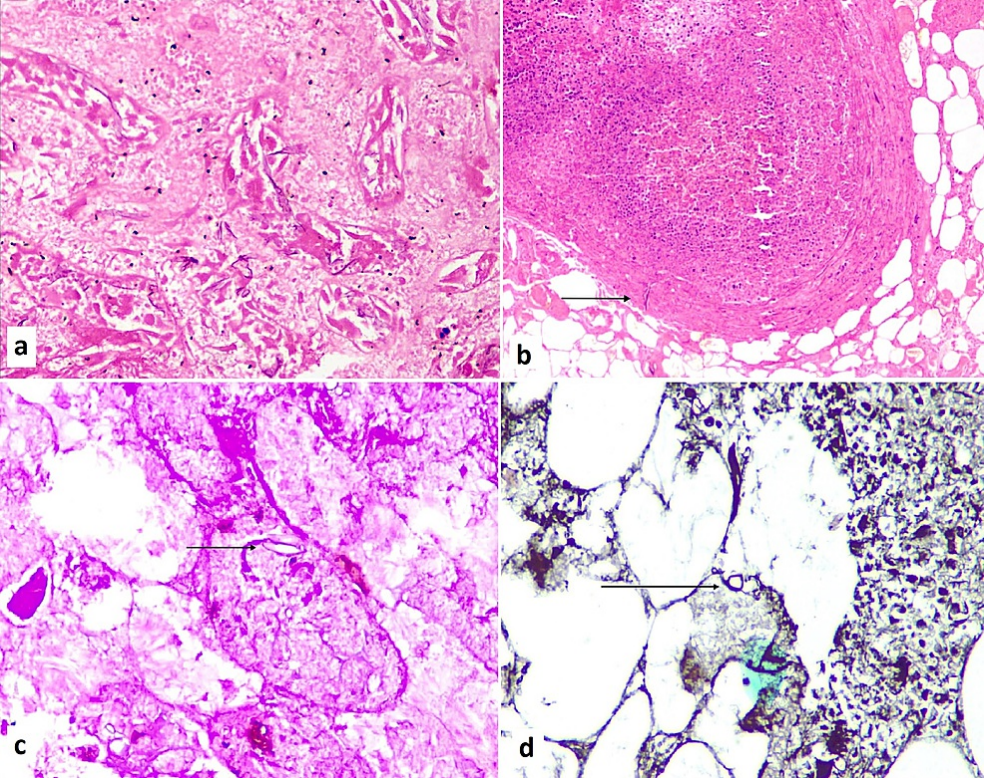
Histopathology images. a) Necrosis and shedding of epithelial cells of renal tubules in the presence of multiple foldable, broad aseptate fungal hyphae of mucor species; b) Mesenteric vein thrombosis and fungal hyphae in the vessel wall (arrow pointed); c) Periodic acid stain positive fungal hyphae (arrow pointed); d) Grocott’s methanamine silver positive fungal hyphae (arrow pointed).

On gross examination of the surgical specimen, the perinephric fat showed chalky deposits and was covered by exudates. The cut surface of the kidney showed discoloration and loss of corticomedullary distinction with medullary congestion. The histopathological examination showed extensive necrosis, dense inflammatory infiltrate, and numerous broad, foldable pauci septate fungal hyphae with right-angle branching confirming the morphology of mucormycosis. The angioinvasion in the segmental vessels was also noted. Similarly, examination of the resected bowel mesentery also revealed angioinvasion of segmental and sub-segmental mesenteric vessels (both arterial and venous) by mucormycosis along with thrombosis leading to gangrene and perforation (Figure 2A-D). Fungal infiltration was limited to the mesentery and not present in the infarcted small intestine.

## Discussion

Mucormycosis is an uncommon and life-threatening fungal infection caused by an omnipresent fungus Rhizopus, belonging to the order *Mucorales*, class Zygomycetes [4]. It is commonly encountered in patients with altered immunity such as patients with uncontrolled diabetes mellitus (DM), post bone marrow or solid organ transplant, lymphoma, leukemia, patients on immunomodulatory drugs, and rarely in immunocompetent patients following trauma or burns, etc. [1, 3, 5].

Recently, there has been a community epidemic of this fatal disease during the current second wave of the COVID-19 pandemic in India. The prime reason hypothesized for promoting the germination of mucor spores includes relative hypoxia, uncontrolled DM or new-onset hyperglycemia due to steroids, diabetic ketoacidosis leading to an acidic medium, high serum iron levels (glycosylation of ferritin and transferrin), and immunosuppressed state with the poor phagocytic activity of white blood cells and macrophages [5-6]. Further, protracted hospitalization, improper use and cleaning of humidifiers, and repeated usage of the contaminated masks are the other probable causes involved in its rapid spread among the general population. In the present case, the patient was on prolonged high-dose steroid therapy, without proper guidance, for a long time after he was diagnosed with COVID-19 infection.

Tissue infarction and necrosis subsequent to the invasion of vessels by fungal hyphae is the hallmark of mucormycosis and these infections are rapidly progressive [5]. It frequently involves nasal sinuses, brain (rhino-orbital-cerebral), lungs, cutaneous/wound infections, kidney, and rarely GI organs [3-5]. GI mucormycosis (GIMM) has been earlier reported predominantly in premature neonates having necrotizing enterocolitis, in neutropenic and immunocompromised adults due to human immunodeficiency virus (HIV) infection and systemic lupus erythematosus [2, 5].

Overall, GIMM accounts for 5%-13% of all cases of mucormycosis, and the majority of cases are diagnosed inadvertently during surgery or post-mortem [7]. The stomach is the most common organ involved in adults followed by the colon while the involvement of the small bowel is rare [2]. The symptoms of GIMM are nonspecific and include abdominal pain, vomiting, abdominal distension, and fever or intra-abdominal abscess. The GI bleeding like hematochezia or melena may also be encountered akin to our case [3].

Pre-operative diagnosis of GIMM remains a diagnostic challenge. Along with the presence of the above-mentioned risk factors, worsening clinical symptoms in spite of broad-spectrum antibiotics and repeated sterile culture of body fluids (like urine and blood) should raise the suspicion of fungal infection [8]. Contrast-enhanced axial imaging of the abdomen can detect indirect signs of mucormycosis like pneumatosis, ischemia, and/or infarction with a lack of any obvious vascular thrombosis [9]. In our case, we could not perform a contrast-enhanced CT scan as he had a deranged renal function test which delayed the diagnosis of mesenteric ischemia.

Direct visualization of aseptate, branching (right angle or acute) fungal hyphae with evidence of giant cell reaction and thrombosis with eosinophilic necrosis of tissue in biopsy or histopathology remain the gold standard for diagnosis [10]. Potassium hydroxide (KOH) mount is used during direct microscopic examination and periodic acid-Schiff (PAS) or Grocott-Gomori's methenamine silver (GMS) stains are utilized during histology to assess the fungal morphology [10].

In our case, non-specific presentation and acute kidney injury along with an extensive intra-abdominal spread with gangrene of duodenum, proximal jejunum, and intermittent areas in ileum and colon were noted due to mucormycosis which, in our knowledge, has not been reported so far. The most likely proposed pathogenesis is the angioinvasive nature of the infection, leading to vascular thrombosis and finally ending up in multiple tissue necrosis [2,5]. Further, in the current case, the involvement of mucormycosis was systemic with involvement of both renal and mesenteric vessels which led to catastrophic complications and eventually patient succumbed to the infection. 

The treatment of GIMM is an emergency and often combines debulking surgery to resect all the infected/necrosed tissue along with antifungal medication and correction of risk factors [4]. Adequate surgical debridement is reported as an independent predictor for favorable outcomes in GIMM. Apart from this, the guidelines recommend long-term treatment with liposomal amphotericin B and posaconazole which have activity against *Mucorales* [11].

## Conclusions

Gastrointestinal mucormycosis is rare and often fatal, and synchronous involvement with renal mucormycosis is further uncommon. Due to the rising incidence of mucormycosis in the current COVID-19 pandemic, one needs to be aware of this rare presentation and maintain a high index of suspicion for its early diagnosis and adequate management. It is more important in diabetic patients who have been treated with corticosteroids and/or immunomodulators. 

## References

[1] Jeong W, Keighley C, Wolfe R (2019). The epidemiology and clinical manifestations of mucormycosis: a systematic review and meta-analysis of case reports. Clin Microbiol Infect.

[2] Spellberg B (2012). Gastrointestinal mucormycosis: an evolving disease. Gastroenterol Hepatol (NY).

[3] Dioverti MV, Cawcutt KA, Abidi M (2015). Gastrointestinal mucormycosis in immunocompromised hosts. Mycoses.

[4] Skiada A, Pavleas I, Drogari-Apiranthitou M (2020). Epidemiology and diagnosis of mucormycosis: an update. J Fungi (Basel).

[5] Pilmis B, Alanio A, Lortholary O (2018). Recent advances in the understanding and management of mucormycosis. F1000Res.

[6] Baldin C, Ibrahim AS (2017). Molecular mechanisms of mucormycosis - the bitter and the sweet. PLoS Pathog.

[7] Kaur H, Ghosh A, Rudramurthy SM (2018). Gastrointestinal mucormycosis in apparently immunocompetent hosts - a review. Mycoses.

[8] Cornely OA, Alastruey-Izquierdo A, Arenz D (2019). Global guideline for the diagnosis and management of mucormycosis: an initiative of the European Confederation of Medical Mycology in cooperation with the Mycoses Study Group Education and Research Consortium. Lancet Infect Dis.

[9] Ghuman SS, Sindhu P, Buxi TBS (2021). CT appearance of gastrointestinal tract mucormycosis. Abdom Radiol (NY).

[10] Chander J, Kaur M, Singla N (2018). Mucormycosis: battle with the deadly enemy over a five-year period in India. J Fungi (Basel).

[11] Brunet K, Rammaert B (2020). Mucormycosis treatment: recommendations, latest advances, and perspectives. J Mycol Med.

